# Mindfulness and music interventions in the workplace: assessment of sustained attention and working memory using a crowdsourcing approach

**DOI:** 10.1186/s40359-022-00810-y

**Published:** 2022-04-27

**Authors:** Johanne Lundager Axelsen, Jacob Stig Jarnot Meline, Walter Staiano, Ulrich Kirk

**Affiliations:** 1 Department of Psychology, University of Southern Denmark, 5230 Odense, Denmark; 2 Department of Physical Education and Sport, University of Valencia, 46010 Valencia, Spain

**Keywords:** Mindfulness, Music, App-based cognitive games, Crowdsourcing, Stress, Sustained attention, N-back

## Abstract

**Background:**

Occupational stress has huge financial as well as human costs. Application of crowdsourcing might be a way to strengthen the investigation of occupational mental health. Therefore, the aim of the study was to assess Danish employees’ stress and cognition by relying on a crowdsourcing approach, as well as investigating the effect of a 30-day mindfulness and music intervention.

**Methods:**

We translated well-validated neuropsychological laboratory- and task-based paradigms into an app-based platform using cognitive games measuring sustained attention and working memory and measuring stress via. Cohen’s Perceived Stress Scale. A total of 623 healthy volunteers from Danish companies participated in the study and were randomized into three groups, which consisted of a 30-day intervention of either mindfulness or music, or a non-intervention control group.

**Results:**

Participants in the mindfulness group showed a significant improvement in the coefficient of sustained attention, working memory capacity and perceived stress (*p* < .001). The music group showed a 38% decrease of self-perceived stress. The control group showed no difference from pre to post in the survey or cognitive outcome measures. Furthermore, there was a significant correlation between usage of the mindfulness and music app and elevated score on both the cognitive games and the perceived stress scale.

**Conclusion:**

The study supports the nascent field of crowdsourcing by being able to replicate data collected in previous well-controlled laboratory studies from a range of experimental cognitive tasks, making it an effective alternative. It also supports mindfulness as an effective intervention in improving mental health in the workplace.

## Background

Occupational stress-related disorder is not only a capricious disease that pertain to health and well-being of individuals. In terms of sick leave, stress-related disorders result in lower productivity and absenteeism from work. Both of which is associated with a substantial financial cost for society [1–3]. WHO has been named stress the “Health Epidemic of the 21st Century” and estimated it to cost American Businesses up to $300 billion a year [4].

Occupational stress research has typically been characterized by employing self-reports methods as the primary outcome metric. However, self-report measures are potentially vulnerable to bias due to a range of factors, including social desirability or response style. Specifically, previous studies suggest that people apply different strategies when completing self-report surveys, which run the risk of producing differences in scores between participants that reflects influences other than item content. This might affect the validity of the causal conclusions for variables in question as it may not be clear precisely which properties is being measured [5–7].

### Lab-based metrics versus app-based metrics

In the domain of occupational mental health there seems to be a need for reformulation of the current reliance on self-reports which provide an impetus to develop other approaches [8]. An alternative approach is to employ research methods using lab-based neuropsychological experimental paradigms, which of course have the textbooks of scientific methodology to lean on in terms of allowing for increased control of the variables of interest. However, such an approach profoundly lacks and changes the ecological validity of carrying out research into occupational stress in larger trials and real-world designs such as the workplace. Instead, assessment of occupational stress in the workplace may be strengthened using novel approaches by adopting insights from the field of crowdsourcing whereby there is a reliance on smartphone data [9–11]. In the nascent field of crowdsourcing, there has recently been attempts at collecting task performance from neuro-psychological experimental paradigms that has been reformulated into small games outside of laboratory settings. Such studies have demonstrated have replicated data collected in well-controlled laboratory studies in experimental tasks from a range of experimental tasks including working memory (i.e. n-back tasks) and go/no-go tasks to mention a few [9, 12–19].

One hypothesis that the current study is investigating, is that employing a crowdsourcing approach using cognitive games may bypass the vulnerability of the status quo in the domain of occupational mental health. A status quo that might come from factors such as social desirability and response bias that are inherent challenges when employing self-reports [20].

As such, this study’s experimental aim was to assess the status of mental health in the workplace among Danish companies by relying on a crowdsourcing approach. To accomplish this aim we employed covert outcome-measures by translating well-validated neuropsychological laboratory- and task-based paradigms into an app-based platform using cognitive games. An advantage was that this approach allowed us to probe cognitive games measuring working memory and response inhibition in a large cohort of participants (N = 623) that would otherwise not be possible to deploy using such tasks in a lab-based context.

A secondary aim was to investigate the effect of *mindfulness* and *music* on cognitive processing and self-reported stress in the workplace among employees in Danish companies. This also contributed to explore and target specific interventions that may promote mental health in the workplace. In the current study we thus aimed to derive cognitive effects (through covert cognitive games using an app-based platform) from two types of interventions in the workplace. The project was carried out by offering employees in Danish companies 30-day training interventions with daily exercises of respectively mindfulness, listening to music or entering the no intervention control group. Participants were randomly selected to receive mindfulness training, music listening for 10 min per day or function as the no intervention control. Both training interventions were performed on app-based platforms.

### An app-based mindfulness intervention in the workplace

As an antidote to stress-induced disorders, it has been shown that mindfulness may be helpful in treatment of pathological and stress-related conditions, and with positive impact on quality of life and well-being [21–28].

A broad range of research has shown that mindfulness is effectful in dampening stress and indeed increase cognitive processing [29–35]. However, it has thus far not been tested whether these salutary cognitive effects are present in an ‘ecological’ context, i.e., in the workplace. Thus, this study aimed to investigate in a large group of Danish employees if listening to music or practicing mindfulness has the same positive effect on increased cognitive processing as when these training regimes were tested in the lab [29–35].

As a behavioral therapy mindfulness seek to improve self-regulation and emotion management through systemic training [36, 37]. Such skills have recently been shown to reduce mind-wandering [29, 34, 38, 39]. Mind-wandering refers to thoughts that are not tied to the immediate task and can be linked with decreased performance on different measures including working memory capacity [40]. Furthermore, recent research suggest that mindfulness training can reduce the effect of mind-wandering and its effect on working memory capacity during high stress [35, 39], enhances attention [41], increases backward digit memory span [42]. Such results points to the fact that mindfulness has positive effects on cognitive processing such as working memory capacity and mind-wandering. In addition, acute stress prompt a shift from relying on more deliberate cognitive processes such as working memory capacity to more automatic processes which presumably is underpinned by subcortical brain structures (for a review, see [43]).

### An app-based music intervention in the workplace

The use of music and specifically concentration and relaxation music in this study aimed to investigate whether relaxation music might affect subject’s ability to increase focus and decrease stress, as well as compare the effects to a mindfulness intervention. Our group have in a previous study shown that both mindfulness and music exhibit effects in terms of enhancing sustained attention compared with a control group [30]. As well as enhancing cognitive control and reduce the detrimental effects on mental fatigue [44]. This result is supported in the literature using music, which has shown significant improvements in attention levels and working memory [45]. Other studies have shown an effect of 30 min of listening to binaural beats in the beta-range on mood and vigilance [46].

### Far transfer effects

In the present study we use cognitive games to measure sustained attention. When comparing mindfulness interventions with music or other types of interventions, one should make a distinction between near and far transfer of skills. Near transfer of skills refer to the improvement within the trained domain where far transfer refers to the generalization of training in one domain to different domains [47]. Yakobi et al. [48] suggest to that working memory and sustained attention, measured in the present study, should be considered a far transfer domain. Far and intermediate transfer effects must be considered smaller and less reliable than near transfer effects. Our cognitive games (SART and n-back task) can be used to investigate far transfer skills such as sustained attention, attention lapse and working memory performance [48].

Previous research on mind wandering has demonstrated that it is associated with a negative effect on performance across several domains including text comprehension [49], increased negative mood [50, 51] and working memory capacity [52]. Interestingly, SART performance has been shown to correlate with everyday attentional failures [53] which highlights the ecological validity of the task.

In this study we wished to extend the current knowledge and address the question if the effects of mindfulness relative to a comparable music intervention on cognitive processing could be captured using app-based metrics.

Specifically, our hypotheses were:

1. The app-based metrics, i.e. the cognitive games would show comparable effects in terms of far transfer skills such as sustained attention relative to validated lab-based metrics such as SART.
2. The mindfulness group would show significant effects over the intervention period, which would be read-out as increasing working memory capacity, increasing sustained attention and decreasing self-reported stress as a function of time (pre vs. post).
3. The non-intervention control group would not exhibit changes in far transfer skills over time (pre vs. post).

## Methods

### Participants

**Fig. 1:**
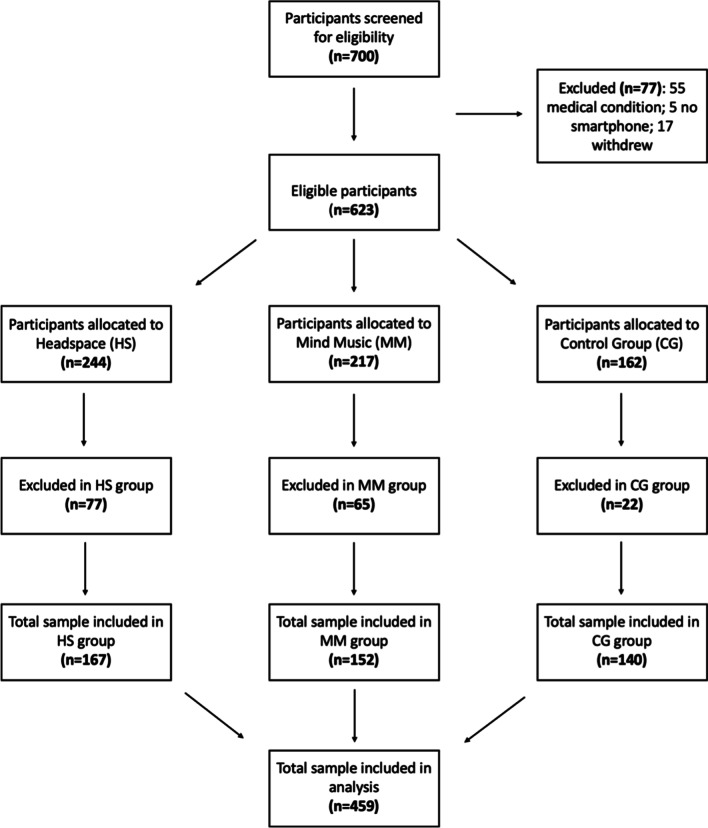
CONSORT flow diagram showing the number of participants in each group and the phases of the study from enrollment to analysis

A total of 623 healthy volunteers participated in the study. Participants were randomized into three groups. 244 participants were allocated to the mindfulness group. 77 participants dropped out of the study either due to missing data points (pre/post measurements) or non-compliance with the app-based home training. Non-compliance was defined as < 20% of the training. The total sample size for the mindfulness group included in the subsequent analysis amounted to 167 participants. The average age of participants in the mindfulness group was 39.5 years (SD ± 9.4; 83 females/84 males). 217 participants were allocated to the music group. 65 participants dropped out of the study either due to missing data points (pre/post measurements) or non-compliance with the app-based training. The total sample size for the music group included in the subsequent analysis amounted to 152 participants. The average age of participants in the music group was 38.5 years (SD ± 9.6; 68 females/84 males). 162 participants were allocated to the control group. 22 participants dropped out of the study due to missing data points (pre/post measurements). The total sample size for the control group included in the subsequent analysis amounted to 140 participants (see Fig. 1). Due to the large sample size in this study, recruitment was performed ad hoc, which made it difficult to control ensure the identical group sizes. The average age of participants in the control group was 38.4 years (SD ± 10.1; 70 females/70 males). All participants in the control group (n = 162) were after study completion given access to either the mindfulness or music app (this information was conveyed to participants in the control group when they signed up for the study). The study was conducted entirely online, and thus all contact with participants was via e-mail. Participant recruitment began in March 2019 and data collection ended in March 2020.

### Recruitment

Recruitment for the current study involved online-based advertisement campaign through the University of Southern Denmark’s Facebook-page and LinkedIn advertisements that solely targeted Small and Medium-sized Enterprises (SME) located in Denmark. Upon interest from the online-campaign, companies were directed to the project website www.etforsoegvaerd.dk, where they received further information about the study. The study was framed as a stress reduction study conducted in the context of the workplace and with the aim to reduce the impact of stress in the Danish working environment. Companies were informed that the study involved either a mindfulness, music or a non-intervention control group lasting 30 days with a required 10 min of daily training using an app-based platform (either mindfulness or music). In addition, companies were informed that their employees would be assigned to one of the three groups in a random manner, which eliminated any self-selection bias across the groups. Sequence generation and randomization was performed by the research team, who were not formally blinded to group allocation. Participants were informed that they would in addition to one of the two intervention *training-apps* get access to a *testing-app* which instructed participants to play brief cognitive games (3–4 min.) and complete surveys during the intervention period. Thus, the entire study was being run using app-based platforms for both interventions and a separate app for collecting outcome measures. Recruitment was explicitly and solely directed at SME’s located in Denmark. It was the companies’ responsibility to recruit participants among their employees for the study. They received standard recruitment material from the research team describing the study in detail which they could distribute among employees in their organization. Each company sent a list of participants/employees who had signed up for the study to the research team after which participants were given specific information about the study logistics and requirements before consenting to participating in the study. This information included that participants at any time during the study had the option to discontinue their participation in the study. Participants were informed that the app-based platforms utilized in the study ran on both Android and IOS, and thus required that participants had access to a smartphone for the study duration. Exclusion criteria were previous experience with mindfulness meditation, and current psychiatric illness or psychiatric medication intake. Participants or companies did not receive monetary compensation for their participation in the study. The study was approved by the local ethical committee (Videnskabsetisk Komité for Region Syddanmark), as well as all procedures and methods were carried out in accordance with the committee’s guidelines and regulations.

### Training app: mindfulness and music

**Fig. 2:**
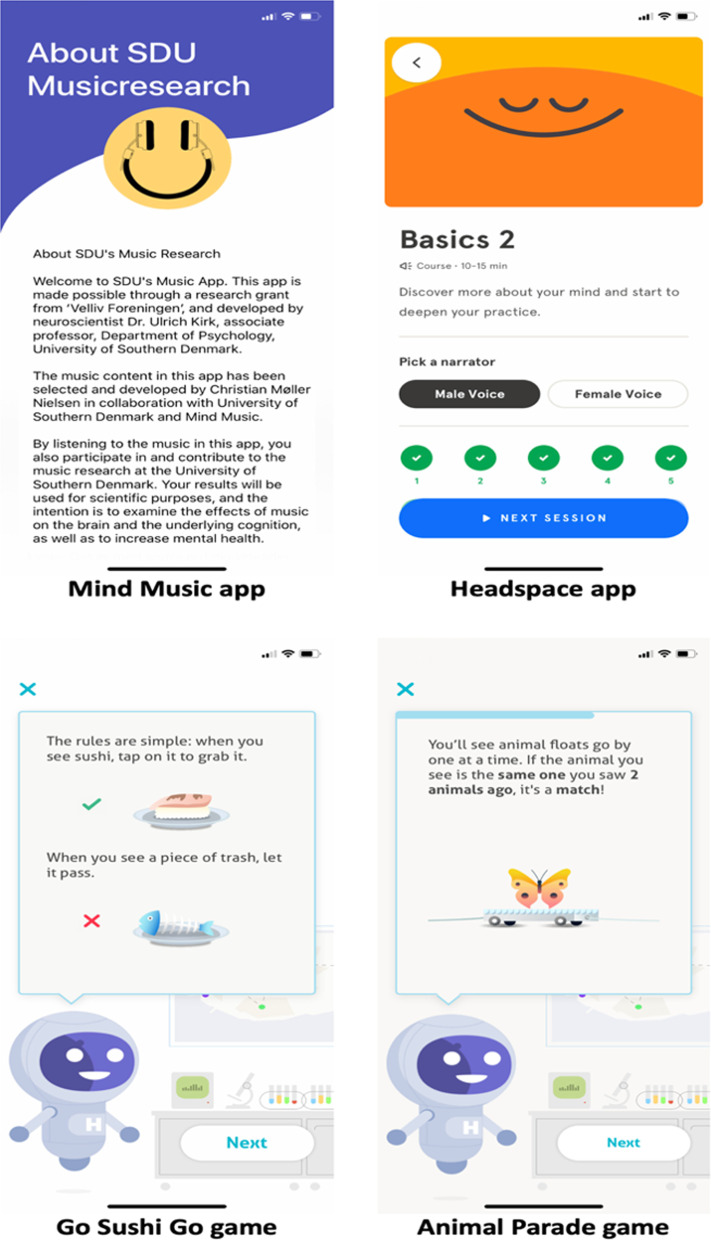
Screenshots of the apps used in the study. Top Left Panel: The music app was custom-made for the study by University of Southern Denmark. Participants were required to listen to instrumental music for 10 min per day for the study duration. Top Right Panel: The Headspace mindfulness training is an app where participants were asked to complete guided audio meditation courses of 10 min duration per day for 30 days. Bottom Left Panel: Instructions for the mind wandering game (Go-Sushi-Go). The narrative of the game was that pieces of sushi were displayed on a conveyor belt and participants were asked to tap the screen as fast as possible (faster reaction time yielded more points) when ‘fresh’ sushi was displayed on the screen and refrain from tapping the screen when pieces of trash appeared on the screen (on these trials participants had to inhibit their response to gain points). Bottom Right Panel: Instructions for the working memory game (Animal Parade). The narrative of the game was that in an animal parade, different pictures of animals were displayed, and participants were asked to make a forced choice whether the animal displayed on the screen is identical or not to the animal displayed 2 trials/animal back

The mindfulness practice intervention consisted of a 30-day app-based program provided by Headspace (https://www.headspace.com/). Similarly, the music intervention was accomplished using a custom-built app-based platform (Fig. 2, upper panels). Participants did not receive an introductory session to mindfulness or music but were provided with written instruction related to installation of the training app and usage for the following 30 days. The content of the Headspace mindfulness training is modelled after the core practices and concept of mindfulness [36]. Specifically, the Headspace group was instructed to follow an introductory course to mindfulness in the app with three levels, namely ‘Basics I–III’, where each level consisted of 10 sessions, or 30 sessions in total. It was not possible to skip sessions and sample the content at will, but they had to follow the session in chronological order. The introductory course (Basics I–III) involved conveying audio-guided introductory and basic principles of mindfulness, as well as guided mindfulness sessions aiming to learn to employ mindfulness techniques such as breath awareness and body scanning. The Headspace app has been applied in various scientific research demonstrating a reduction in mind-wandering [29, 30], as well as self-reported stress [54], and an increase in self-reported well-being [55, 56] and self-reported mindfulness [57, 58]. The daily training requirement was 10 min. corresponding to completion of one session in the course ‘Basics I–III’ per day for the duration of the study intervention.

By contrast, the music group was instructed to listen to instrumental music available in the app. The music was organized according to different playlists or headlines in the app, specifically ‘focus’, ‘binaural beats’, ‘piano’ and ‘Lo-Fi’. Each of the 4 playlists consisted of 30 tracks of various length. Participants were instructed to freely select which playlists to listen to and they were free to listen to any or all 4 playlists during the study. The music app was built for the purpose of the current study. The daily listening requirement was 10 min. for the music group to mimic and compare across the mindfulness intervention group.

Participants in both app-based intervention groups were instructed to follow the programs in full and complete the daily training/listening requirements at any time during the day that fitted with their schedule. The usage of the apps was tracked using the timestamps provided in the Headspace app and music app. Usage information was available to participants to keep track of their daily usage during the study. The timeseries containing each practice session for each participant was extracted from the apps upon completion of the study and was processed for further analysis.

### Testing app: collecting outcome measures

To accommodate the experimental aims of employing primarily covert outcome measures (using cognitive games) to probe cognitive and mental health among a large cohort of employees in Danish SME’s, we had an external developer build the cognitive games app for smartphone (https://www.datacubed.com/). This approach of designing user-engaging and brief cognitive games while measuring key cognitive processes such as performance of working memory (n-back task) and sustained attention (go/no go task) was essential to probe the large sample-size outside of a laboratory-based environment (Fig. 2, lower panels). In addition, it was essential to ensure comparison between cognitive games performance and traditional valid neuropsychological laboratory- and task-based paradigms.

To maintain motivation in using the app among our participants, the app was designed such that participants was set up with an avatar in the app. Completion of tasks and survey in the app resulted in that participants earned various amounts of points based on performance. Point could subsequently be used to buy a range of items for their avatar in the app or gain access to new worlds within the app. This feature was made available with the hope to maintain interest (user-engagement) in the app for the entire duration of the study.

Participants were instructed to play the games 3 times and complete the games within a time-window of 3 days prior to the intervention start, and in addition complete surveys that was also embedded in the app in a similar time-window. Following the 30-day intervention, participants were notified to complete the post-measure comprising the identical metrics as used at the pre-intervention time-point. Participants were reminded to complete the assignments through notifications on the smartphone and email-reminders to participants who were at risk of not complying with these instructions (as gauged from the app dashboard where the research team had access to compliance percentage of all participants enrolled in the study). The participants could complete the games at any time of day but were instructed to complete the games when there were not too many distractions around them so they could focus on the task.

### N-back game/working memory game (‘animal parade’)

In the working memory (WM) game, participants were asked to remember and report the identity of animals that appeared in a parade. Participants saw one animal at a time in a parade. Every animal that appeared constituted a trial. Each trial ended with a forced choice option of indicating whether the animal displayed on any given trial in the parade was a ‘match’ or ‘not a match’ (i.e. identical) to the animal that appeared two trials back (2-back task). The participants received feedback on each trial if the response was correct or incorrect. The progression of the game was such that participants were initially presented with a few practice trials, which was followed by 3 sessions with each 34 trials comprising a total of 102 trials. There was various information extracted from the game, specifically hit rate (number of correct responses) and reaction-time, but here we focus on the former. The hit rate in this 2-back game was computed as the absolute number of correct trials averaged across the 3 repetition of the game (completed on 3 consecutive days), that is each participant completed 102 trials × 3 repetitions comprising a total number 306 trials that was averaged for each participant. WM performance was determined by calculating the percentage from the total number of trials completed and number of correct responses (i.e. accuracy scores). The test–retest reliability for the n-back task (2-back) on accuracy was r = 0.538 and on reaction time it was r = 0.691 when measured on two occasions in healthy subjects [59].

### Sustained attention game (‘Go Sushi Go’)

Laboratory evidence of attention lapse is frequently captured by the Sustained Attention to Response Task (SART) [53]. Robertson et al. [53] defines ‘sustained attention’ as “*as the ability to self-sustain mindful, conscious processing of stimuli whose repetitive, non-arousing qualities would otherwise lead to habituation and distraction to other stimuli*” (pp. 747). Sustained attention can be measured by the SART. Specifically, the SART is a go/no go task that require that go-trials are more frequent and thus creates a habitual response pattern that must be periodically overwritten by the more infrequent no-go trials which require that the participant refrains from responding. Thus, the critical measure of ‘sustained attention’ yields a count of the success rate to withhold a response when presented with infrequent trials. The SART has shown good reliability in the error score between two occasions measured on healthy subjects were the Pearson correlations was r = 0.76, showing that performance on this test is stable over time [53].

In the attention lapse game, the narrative that participants were presented with was that pieces of sushi would be displayed on a conveyor belt appearing a rapid succession. There were two trial conditions: one in which a fresh piece of sushi appeared on the conveyor belt/screen and another in which a bad piece of sushi (i.e. trash-conditions) appeared on the screen. The two conditions were visually distinct. Participants were instructed to tap the screen only when fresh sushi appeared and not tap the screen (‘no go’ trials) when trash-conditions (i.e. outdated pieces of sushi) appeared. The trash-conditions were under-sampled such that out of a total of 96 trials, there were 20 no-go trials. The participants received feedback on each trial displayed if the response was correct or incorrect. The progression of the game was such that initially participants were presented with a few practice trials, which was followed by 2 sessions with each 48 trials comprising a total of 96 trials. There was various information extracted from the game, specifically hit rate (number of correct responses) and reaction-time, but here we focus on the former. The hit rate in this go/no-go game was computed as the absolute number of correct trials averaged across the 3 repetition of the game (completed on 3 consecutive days), that is each participant completed 96 trials × 3 repetitions comprising a total number 288 trials that was averaged for each participant. The sustained attention coefficient was determined by calculating the percentage of hit rates during the no-go trials (i.e. %nogo success).

### Psychological measures

We employed the Perceived Stress Scale (PSS) [60] using the app-based platform as mentioned above that also ran the cognitive games on smartphone. The PSS is a 10-item scale designed to measure the perception of stress and has shown good reliability with Cronbach’s Alpha between 0.6 and 0.85 [61]. Initially, all participants were asked to complete the PSS, and again after the intervention.

### Statistical analysis

We report how we determined our sample size, all data exclusions (if any), all manipulations, and all measures in the study [62].

All data is presented in mean ± SD unless otherwise stated.

Assumptions of normal distribution and sphericity of data were checked accordingly. Greenhouse–Geisser correction to the degrees of freedom was applied when violations to sphericity were present.

Mixed 2 × 3 ANOVAs were used to assess if there were differences pre and post intervention on the groups’ performance on the cognitive games and PSS. Significant interaction effects from the mixed ANOVA were followed up with *t* tests. Significance was set at 0.05 (2-tailed) for all analyses.

Pearson correlation analysis was conducted to investigate correlations between cognitive game performance and training dose–effect of the two active interventions (mindfulness and music). Pearson correlations (R) were considered small = 0.1, medium = 0.24 and large = 0.37 suggested by Cohen [63]. The effect sizes for mixed measures ANOVAs were calculated as partial eta squared (*η*^*2*^*p*), using small = 0.02, medium = 0.13 and large = 0.26 interpretation for effect size [64]. The effect sizes for the *t* tests were calculated as Cohen’s *d* using small = 0.2, medium = 0.5 and large 0.8 suggested by Cohen [64].

All data analysis was conducted using the statistical packages for social science (SPSS version 26).

## Results

Table 1 shows mean and standard deviations for the mindfulness, music and control group on the PSS, Go Sushi and Animal Parade measured at baseline and after the 30-day intervention.

**Table 1:** Mean (± standard deviation) for the three groups on the three outcome measures at baseline (T1) and after the 30-day intervention (T2)

	Mindfulness group	Music group	Control group
PSS *baseline*	16.21 ± 9.17*	14.99 ± 8.27*	15.99 ± 8.57
PSS *post*	9.16 ± 5.5*	9.2 ± 5.04*	14.38 ± 6.38
GoSushi *baseline*	49.31 ± 24.36*	50.59 ± 21.43*	50.52 ± 17.15
GoSushi *post*	69.63 ± 19.07*	58.38 ± 22.38*	54.11 ± 19.49
Animal Parade *baseline*	82.5 ± 5.26*	82.96 ± 5.58	83.31 ± 5.79
Animal Parade *Post*	88.16 ± 5.59*	83.15 ± 6.19	83.22 ± 6.63

The * next to the mean is a significant Pearson correlation between the cognitive game performance and training dose–effect (only measured of the two active interventions (mindfulness and music))

^*^Correlation is significant at the 0.01 level (2-tailed)

### PSS results

**Fig. 3:**
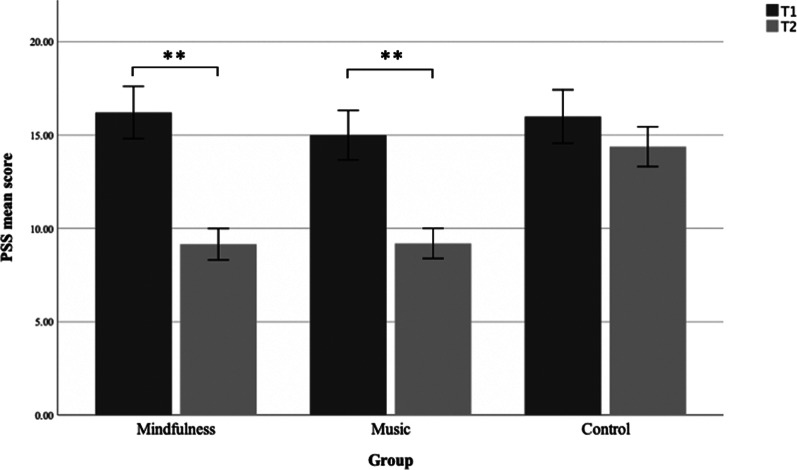
PSS mean. PSS mean score pre (T1) and post (T2) the intervention for the mindfulness, music and control group. Error bars are 95% CI. Significance bars show the significant difference of the mindfulness’ and music group’s PSS-score from T1 to T2. There was no difference between the control group’s PSS-score on T1 and T2

To assess if there were differences on perceived stress, as measured by the PSS, at baseline (T1) relative to after the interventions (T2), we employed a mixed 2 × 3 ANOVA to inspect time (T1, T2) and group/intervention type (mindfulness, music, control) (see Fig. 3). A significant group × time interaction for PSS was found (*F*(2, 455) = 11.74, *p* > 0.001, η^2^p = 0.05). Follow up paired *t* test revealed that in the control group there were no significant changes in the PSS-score from baseline to post measurement (paired *t*(139) = 1.925, *p* = 0.06). However, in the mindfulness group (paired *t*(166) = 8.644, *p* < 0.001, *d* = 0.67) and the music group (paired *t*(151) = 7.33, *p* < 0.001, *d* = 0.6) there was a significant decrease in the PSS-score from baseline to post measurement indicating significant lower stress.

### Go Sushi Go game

**Fig. 4:**
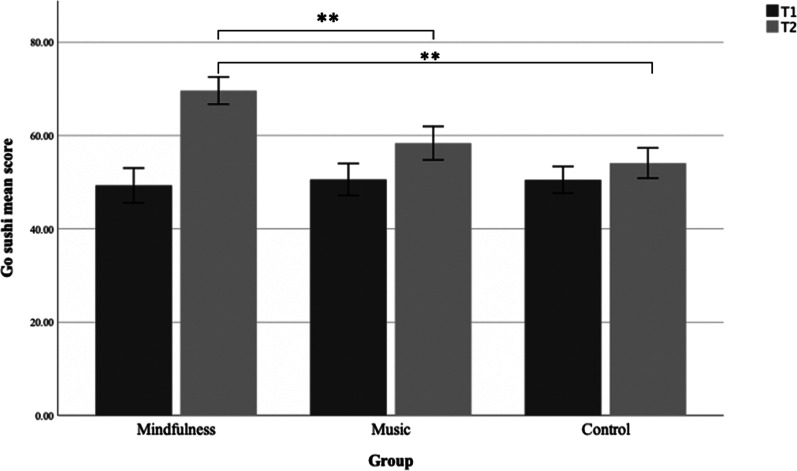
Go Sushi mean. Go Sushi mean score from pre (T1) to post (T2) intervention for the three groups. Error bars are 95% CI. Significance bars show the significant difference between the mindfulness group and the music group, as well as the mindfulness group and the control group on T2

To assess if there were performance differences in the sustained attention as measured by the GoSushi game at baseline (T1) relative to after the interventions (T2), we employed a mixed 2 × 3 ANOVA to inspect time (T1, T2) and group/intervention type (mindfulness, music, control) (see Fig. 4). A significant group × time interaction for GoSushi was found (*F*(2, 456) = 17.965, *p* > 0.001, η^2^p = 0.073). Follow up paired *t* test revealed that in the control group there were no significant changes in the GoSushi-score from baseline to post measurement (paired *t*(139) = − 1.640, *p* = 0.1). However, in the mindfulness group (paired *t*(166) = − 10.374, *p* < 0.001, *d* = − 0.8) and the music group (paired *t*(151) = − 3.621, *p* < 0.001, *d* = − 0.3) there was a significant increase in the GoSushi-score from baseline to post measurement indicating significant higher performance on the GoSushi-game.

A one-way between groups ANOVA was conducted to explore differences in performance at T1 and T2 across the groups on the GoSushiGo task. There was no significant difference in performance on T1 between the three groups: F (2, 458) = 0.18, *p* = 0.836. On T2 there was a significant difference between the group’s performance on the GoSushi task: F (2, 458) = 24, 30, *p* < 0.001. As the variance between the groups were equal and the group sizes were different, post hoc Hochberg’s GT2 tests were used to inspect differences between the groups at phase 4. Hochberg tests indicated that there was a significant difference between the mindfulness group (M = 69.63, SD = 19.1) and both the control group (M = 54.11, SD = 19.49) and the music group (M = 58.38, SD = 22.38).

### Lab-based versus app-based metrics (Go Sushi Go)

We first assessed if the degree of sustained attention as measured through %nogo success in the GoSushi was similar to a comparable laboratory-based study [34], which reported %nogo success (or target accuracy) of 52.2% (± 21.8%) for 18 control subjects at baseline and 44.5% (± 21.7%) for 24 subjects at baseline in a treatment group. The variance (SD) across the laboratory sample and the app-based sample was comparable. The effect size in our Mixed Anova (interaction effect) was considerably lower than that collected in a similar task under the laboratory conditions (η^2^p = 0.24), although our effect size in the paired comparison in the mindfulness group showed a higher effect size (d = − 0.8) than the mental training group in the laboratory study (d = 0.31). Participants in the laboratory study each completed 546 trials of which 163 were practice trials yielding a total of 383 trials included in the analysis. In our app-based study we included a total of 288 trials for each participant in the analysis. This point as well as the uncontrolled environment in which the task was performed could be reflecting the difference in effect size [9].

### Animal Parade game

**Fig. 5:**
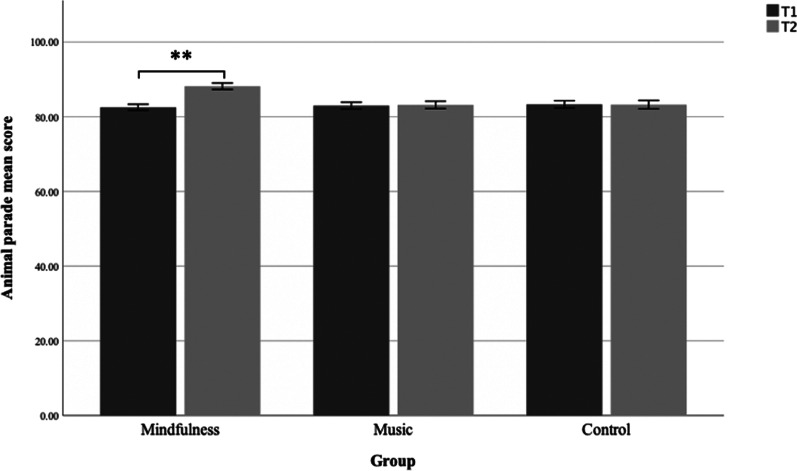
Animal parade mean. Animal parade mean score from pre (T1) to post (T2) intervention for the three groups. Error bars are 95% CI. The mindfulness group had a greater improvement from pre to post intervention than the music group and control group (*p* < 0.001)

To assess if there were performance differences in accuracy scores on the Animal Parade game (AP) at baseline (T1) relative to after the interventions (T2), we employed a mixed 2 × 3 ANOVA to inspect time (T1, T2) and group/intervention type (mindfulness, music, control) (see Fig. 5). A significant group × time interaction for AP was found (*F*(2, 456) = 46.556, *p* > 0.001, η^2^p = 0.170). Follow up paired *t* test revealed that in the music group (paired *t*(151) = -0.429, *p* = 0.67) and control group (paired *t*(139) = 0.160, *p* = 0.87) there were no significant changes in the AP-score from baseline to post measurement. In the mindfulness group there was a significant increase in the AP-score from baseline to post measurement (paired *t*(166) = − 12.31, *p* < 0.001, *d* = − 0.95) indicating significant higher accuracy-performance on the AP-game.

A one-way between groups ANOVA was conducted to explore differences in performance at T1 and T2 across the groups. There was no significant difference in performance on T1 on the animal parade task between the three groups: F (2, 458) = 0.826, *p* = 0.443. On T2 there was a significant difference between the group’s performance on the animal parade: F (2, 458) = 35.05, *p* < 0.001. As the variance between the groups were equal and the group sizes were different, post hoc Hochberg’s GT2 tests were used to inspect differences between the groups at phase 4. Hochberg tests indicated that there was a significant difference between the mindfulness group (M = 88.16, SD = 5.59) and both the control group (M = 83.22, SD = 6.63) and the music group (M = 83.15, SD = 6.19).

### Lab-based versus app-based metric (Animal Parade)

The performance on the AP game as measured through accuracy scores was similar to a comparable laboratory-based study [65], which reported accuracy scores on a 2-back task of 83.9% (± 7.6%) for 17 control subjects. The variance (SD) across the laboratory sample and the app-based sample was also comparable. Participants in the laboratory study each completed 3 blocks of the 2-back task with each block consisting of 25 trials comprising a total of 75 trials. In our app-based study we included a total of 306 trials for each participant in the analysis. A second study investigating performance on the n-back task after a mindfulness meditation course in a controlled setting found a higher effect size on a Mixed Anova on the interaction between group and intervention (η^2^p = 0.25) [66].

### Usage

The total usage of the mindfulness app and the music app was calculated for each group. The mindfulness group had a mean usage of 178.7 min and the music group had a mean usage of 195.3 min. To test if there was a significant difference between the two groups’ usage an independent samples t-test was conducted. There was a significant difference in mean usage between the mindfulness group and the music group (*t*_308.632_ = − 4.19, *p* < 0.001, Cohen’s d = 0.489 [95% CI 0.265–0.711]). The music group had an average usage time of the music app that was 16.6 min higher than the mindfulness groups’ usage of the Headspace app.

### Correlation between training app usage and cognitive games app

The relationship between usage, as measured by time used on the app in minutes and outcome, as measured by GoSushi score, (difference in score) was investigated using the Pearson product-moment correlation coefficient (see Table 2). Preliminary analyses were performed to ensure no violation of the assumption of normality, linearity and homoscedasticity. There was found a small, but significant correlation between the usage and performance variables, *r*(457) = 0.14, *p* = 0.002. There was no significant difference between the mindfulness group and the music group, *p* = 0.33 (mindfulness *r*(165) = 0.014, *p* = 0.87 and music, *r*(150) = − 0.12, *p* = 0.12).

**Table 2:** Pearson correlation coefficient of PSS score, GoSushi difference score Animal Parade difference score and app usage

	App usage
PSS	
GoSushi	r = 0.147*
Animal Parade	r = 0.191*

^*^Correlation is significant at the 0.01 level (2-tailed)

The relationship between usage, as measured by time used on the app in minutes and outcome, as measured by the Animal Parade accuracy score (difference in score) was investigated using the Pearson product-moment correlation coefficient (see Table 2). Preliminary analyses were performed to ensure no violation of the assumption of normality, linearity and homoscedasticity. There was found a small, but significant correlation between usage and performance, *r*(457) = 0.191, *p* < 0.001. There was no significant difference between the mindfulness group and the music group, *p* = 0.57 (mindfulness *r*(165) = 0.23, *p* = 0.002 and music, *r*(150) = − 0.171, *p* = 0.035).

## Discussion

In the current study, we demonstrated an effect of mindfulness practice on perceived stress, mind wandering and working memory in ecological settings (i.e. the in workplace) using a crowdsourcing approach over a period of 30 days. Specifically, a daily 10-min usage of a mindfulness app over the 30-day period, resulted in a 43% reduction in perceived stress, 20% improvement in sustained attention, and a 6% improvement in working memory performance, significant at the alpha level of 0.05. Perhaps surprisingly, the current study showed that our active comparison intervention, namely music, also exhibited a significant improvement on perceived stress. Listening to 10 min of music daily for 30 days, resulted in a 38% decrease on subsequent self-perceived stress. The non-intervention control group did not result in differences, but as expected, maintained performance on the perceived stress scale (PSS), attention lapse task, and working memory task across the intervention period. As such, the experimental aim in the current study was an exploration of the status of mental health in the workplace among Danish companies by relying on a crowdsourcing approach. To accomplish this aim we employed covert outcome-measures by translating well-validated neuropsychological laboratory- and task-based paradigms into an app-based platform using cognitive games.

An advantage was that this approach allowed us to probe cognitive games measuring working memory and response inhibition in a large cohort of participants (N = 623) that would otherwise not be possible to deploy using such tasks in a lab-based context. We argue that the improvement on the SART can be transferred to actual workplace attention as studies support the view that SART is indeed a measure of sustained attention as performance on it is predicted by sustained attention. The SART has shown internal consistency when tested for reliability by Robertson et al. [53], the authors found that SART performance correlates with self-reports of attentional and other ‘cognitive failure’ in everyday life and therefore the workplace [53]

### App set-up

The field of workplace stress calls for a reformation of the current use of self-report methods to investigate workers’ mental health. Lab-based neuropsychological experimental paradigms are a way to objectively measure stress and performance on cognitive tasks. In this study we demonstrate that smartphone apps can provide a reliable measure of workplace stress, that is smartphone apps allow for more reliable longitudinal and cross-study data-collection as well as data from multiple timepoints [9]. We had an external developer design the cognitive games app, with focus on it being user-engaging and -friendly. The games had to be brief but also measure key cognitive processes such as working memory performance and sustained attention, this made it possible to compare the game performance with traditional well-validated neuropsychological laboratory- and task-based paradigms used in other studies. Both the animal parade game and the sushi game were comparable with results from laboratory-based studies by Morrison et al. [34] and Miller et al. [65].

### Validity of crowdsourcing

The fact, that we were able to detect a significant effect of mindfulness on all levels, supports that mindfulness training have an effect, and that this is more than an acute effect. This also supports the validity of our app-based approach, when testing the mindfulness group’s stress level on the PSS, their far transfer skills. Furthermore, when one compares crowdsourcing with lab-based experiments, crowdsourcing has some clear advantages. It has been shown to be very effective and might be a better and more obtainable alternative in some instances. It is easier to obtain a larger sample size, than that of a lab-based experiment, due to it being less time consuming and expensive, since we do not require human interaction every time a test is being performed [9]. By enabling the participant to use their device to be tested, there is no need for them to show up at a laboratory, making the crowdsourcing approach less intrusive as well [11]. The fact, that the human interaction is lessened, also removes the possibility of the experimenter unintentionally cueing the wished response and the participants might be less subject to experimental biases such as demand characteristics [66]. We are also able to bypass the use of questionnaires, except for the PSS. The potential disadvantage of questionnaires and self-reporting in general is that they, as all forms of testing, are not perfect when it comes to validity and reliability. Social desirability is one of the larger issues, which can be avoided, when crowdsourcing relies on cognitive task performance.

### Mindfulness intervention effects

Our results support the hypothesis that a daily 10-min mindfulness intervention over a period of 30 days, would decrease perceived stress, measured on the PSS, improve sustained attention, understood as the ability to withhold a prepotent response during a monotonous task, measured by go/no go task, and increase working memory performance, measured by an n-back task. These results are consistent with previous findings on mindfulness’ effects on mind wandering and stress [29, 30, 44]. This study also contributes to the understanding, that whether the intervention is an 8-week MBSR course, 4-week usage of a mindfulness app, or a 10-min on the spot exercise, it has a salutary effect on sustained attention [40, 44, 67, 68].

The fact, that we found a significant effect of the music intervention on sustained attention is in line with previous studies [30, 44]. This effect was also found in a lab-based setting and non-ecological setting, albeit an acute effect. We did not, however, find a significant effect on working memory in the animal parade n-back task. Our ecological setting might explain that we were unable to find the effect on working memory, in that we in the current study did not measure acute effects, but rather whether an entrainment effect was present or not. This study then seems to support, that music does have an entrainment effect on the far transfer skills such as sustained attention, but not on working memory, but further research is required.

Music does however seem to have an entrainment effect on perceived stress. This does not exclude, that music might have an effect on far transfer skills, if implemented shortly prior to the test, as previous studies have found it enhancing cognitive focus and, in some cases, working memory capacity [46, 69]. Music might help the individual to focus on the task at hand and make the individual less likely to be “lost in thoughts”. The music-intervention however may require to be an acute and on-the-spot intervention, where mindfulness helps the individuals’ focus when facing future challenges and strengthens the working memory performance in a more general way. Mindfulness might improve metacognitive regulation, and therefore increase the individual’s awareness of mind-wandering [44]. Robinson et al. [70] investigated whether binaural beats could be an effective way of improving sustained attention. The authors did not find an effect of binaural beats on sustained attention which align with our results that the music group did not improve their performance on the cognitive games after the 30-days intervention. It could be that auditory interventions such as music and especially beta-frequency binaural beat stimulation is not powerful enough to argument sustained attention and cognition in general. The reason why mindfulness training shows an entrainment effect could be, that it offers strategies to better handling stressful situations and how to economize mental energy [71]. In support of this, work from our group have recently shown that mindfulness has a more chronic effect on the cardiovascular parameter heart rate variability (HRV) as the effect on HRV was pronounced during day- and nighttime when no formal mindfulness was taking place [72]. The improvement in HRV was only present for the active-control music-group during the daily music sessions, again pointing to the fact that music has an acute effect, also on the psychophysiological level.

### Usage

We found a positive correlation between usage and cognitive tasks, indicating, that the more mindfulness sessions completed, the bigger the effect on far transfer skills. These effects are supported by previous research which has demonstrated similar effects using on 2 weeks of daily mindfulness training [35]. There is also evidence that experienced mindfulness users are less affected by mental fatigue than a novice mindfulness group [44] pointing to the fact that the quantity mindfulness practice has significant effects on cognitive control.

### Limitations

We acknowledge that the sample size in each of the 3 groups was not entirely balanced. Due to the large sample size (N = 623) it was logistically difficult to ensure that sample size was identical in each group. This may have been a limitation in the inferences made. However, we note that drop out ensured that the total sample included was comparable across the 3 groups. We have conducted sensitivity analysis to investigate what effect size the study would be powered to detect. A mixed between-subjects ANOVA with 459 participants across three groups and two measurements would be sensitive to effects of η^2^p = 0.016 with 80% power (alpha = 0.05). This means the study would not be able to reliably detect effects smaller than η^2^p = 0.016. An independent samples t-test with the mindfulness group (n = 167) and the music group (n = 152) would be sensitive to effects of Cohen’s d = 0.28 with 80% power (alpha = 0.05, two-tailed). This means the study would not be able to reliably detect effects smaller than Cohen’s d = 0.28.

One criticism might be that participants in the mindfulness training condition simply experiencing a placebo effect because they knew they were in a mindfulness group and because of the general belief that mindfulness is beneficial. However, we argue against this possibility in that we found a correlation between usage (time spent on the app) in both the mindfulness and music group and performance on both cognitive games, which suggest that indeed the enhanced cognitive effect was a function of time spent in app.

Concerning the present study’s cognitive tasks, one should be aware that we have only focused on a single dependent variable in the SART and n-back task. Also, the results should be considered with caution as the task could reflect other factors such as motivation, distraction etc. As the tasks, used in the current study, measure far transfer effects of mindfulness and music, we point to the fact that these skills might be less reliable than near transfer effects.

## Conclusion

This study provides important insights into the effectiveness of employing crowdsourcing in the field of occupational mental health. We did this, by employing covert outcome-measures by translating well-validated neuropsychological laboratory- and task-based paradigms into an app-based platform using cognitive games. This approach empowered us to investigate the effect of 30-day mindfulness or music intervention on perceived stress, sustained and working memory performance in a large cohort of participants (n = 623). The results showed that a daily 10-min usage of a mindfulness app resulted in a 43% reduction in perceived stress, 20% improvement in sustained attention, and a 6% improvement in working memory performance. Perhaps surprisingly, the music intervention also exhibited a significant improvement on perceived stress with a 38% reduction. The non-intervention control group did not result in differences across the intervention period. This study’s results have two important conclusions: (1) crowdsourcing can be compared to data collected in well-controlled laboratory studies from a range of experimental tasks, making it an effective alternative, and (2) mindfulness seems to an effective intervention in improving mental health among employees. Future studies may consider employing crowdsourcing as a way collect cognitive performance data and assess mental health using more objective variables relative or in addition to self-reports in the domain of occupational mental health.

## Data Availability

The datasets generated and/or analyzed during the current study are not publicly available due to anonymity of the participants but are available from the corresponding author on reasonable request.
